# Research Advances in Cancer-Associated Fibroblasts in Prostate Cancer Progression

**DOI:** 10.3390/biom15101369

**Published:** 2025-09-26

**Authors:** Zhonghao Tang, Si Shen, Chenwei Gu, Sixin Li, Yan Qin, Yuanyuan Mi

**Affiliations:** 1 Department of Urology, Affiliated Hospital of Jiangnan University, 1000 Hefeng Road, Wuxi 214122, China; 2 Wuxi School of Medicine, Jiangnan University, 1800 Lihudadao, Wuxi 214122, China; 3 Department of Pathology, Affiliated Hospital of Jiangnan University, 1000 Hefeng Road, Wuxi 214122, China

**Keywords:** prostate cancer, cancer-associated fibroblasts, tumor microenvironment, targeted therapy

## Abstract

The tumor microenvironment (TME) is crucial for tumor growth and progression, within which cancer-associated fibroblasts (CAFs) play a central role in regulating cancer cell proliferation, metastasis, and therapy resistance through various mechanisms. Although early-stage prostate cancer (PCa) has a high cure rate, advanced disease often becomes difficult to manage due to resistance to standard therapies such as androgen deprivation therapy (ADT). Therefore, a deep understanding of the interaction mechanisms between CAFs and PCa cells is essential for developing novel therapeutic strategies targeting resistant advanced PCa. This review systematically summarizes key signaling pathways and molecular mechanisms through which CAFs promote PCa progression, as recently discovered, evaluates the potential of CAFs as prognostic biomarkers, and discusses novel CAF-based therapeutic targets and intervention strategies for PCa.

## 1. Introduction

Prostate cancer (PCa) is the second most common cancer and the fifth leading cause of cancer death among men globally. While incidence and mortality rates for PCa have been declining or stabilizing in most countries, they continue to rise in several nations across Africa, Asia, Latin America and the Caribbean, as well as Central and Eastern Europe. This trend may be attributed to two key factors: on one hand, enhanced detection rates have led to increased identification of cases, thereby elevating reported incidence; on the other hand, constrained access to treatment and limited healthcare resources likely contribute to higher mortality [1].

PCa can be classified as androgen-sensitive or androgen-insensitive based on its response to androgens, a distinction that directly influences tumor response to testosterone stimulation and potential treatment strategies [2]. Specific treatment strategies depend on factors such as tumor characteristics, PSA levels, pathological grade and stage, and recurrence risk. For instance, low-risk PCa is typically managed with radical prostatectomy (surgical removal of the prostate and surrounding tissues) or radiotherapy [3]. For cases extending beyond the prostate or recurring, androgen deprivation therapy (ADT, also known as hormone therapy) is recommended [4]. ADT is indicated for patients with biochemical recurrence after definitive initial treatment, locally advanced disease, or metastatic disease, providing initial benefits. However, most patients progress to castration-resistant prostate cancer (CRPC) within 2–3 years of ADT initiation [5]. CRPC, formerly termed hormone-refractory PCa, is now defined as disease progression despite medical or surgical castration (serum testosterone at castrate levels) [6].

The tumor microenvironment (TME) is a complex ecosystem comprising vascular networks, the extracellular matrix (ECM), various signaling molecules, and non-tumor cells including immune cells, fibroblasts, and adipocytes, each playing distinct roles [7]. Within this milieu, tumor cells engage in complex interactions with both immune and non-immune cells, collectively driving pro-tumor or anti-tumor effects [8]. Fibroblasts, mesenchymal-derived stromal cells, are key to forming connective tissue by secreting collagen and maintaining normal tissue architecture. Quiescent fibroblasts can be activated by stimuli like inflammatory cytokines, playing vital roles in wound healing and tumorigenesis [9]. It is widely accepted that fibroblasts located within or adjacent to tumors undergo a characteristic transformation into activated cancer-associated fibroblasts (CAFs) in response to TME signals, forming the basis of the “cancer-associated” designation [10].

In contrast to other solid tumors, primary prostate cancer exhibits a unique metabolic profile characterized by reduced dependence on glycolysis and greater reliance on oxidative phosphorylation (OXPHOS) for energy production [11]. However, upon progression to advanced and treatment-resistant stages such as CRPC, its metabolic phenotype undergoes significant reprogramming, shifting toward increased dependence on choline metabolism, amino acid metabolism, and glycolysis to support tumor growth and progression [12]. Throughout this process, CAFs play a central role in shaping the metabolic microenvironment of prostate cancer. During early tumor stages, CAFs support OXPHOS in cancer cells by supplying glycolytic metabolites; in later stages, they further promote metabolic plasticity and invasive behavior. CAFs exhibit a form of metabolic “self-sacrifice” in the so-called “reverse Warburg effect”: they sustain high glycolytic activity to provide energy-rich substrates for tumor cells, thereby supporting their high demands for oxidative metabolism and biosynthetic processes [13].

## 2. Impact of CAFs on PCa Progression

CAFs promote PCa cell proliferation, metastasis, and therapy resistance through their critical functions within the TME [14]. CAFs drive tumor progression, metastasis, and therapy resistance by inducing reversible DNA methylation reprogramming—specifically, through DNMT3A-mediated promoter hypermethylation and global hypomethylation—to regulate key genes (such as ZEB1, GRHL2, and CD44), thereby promoting EMT and maintaining prostate cancer stem cell properties [15]. CAFs are a key cellular component of the reactive stroma in prostate cancer, and their abundance and activation status correlate strongly with Gleason score. In Gleason grade 3 lesions, the reactive stroma consists of approximately 50% fibroblasts and 50% myofibroblasts. In Gleason grade 4 lesions, the stroma is predominantly composed of myofibroblasts. Tumors with high Gleason scores (grade 4 and above) exhibit more extensive reactive stroma, higher numbers of CAFs/myofibroblasts, and increased expression of pro-tumorigenic factors such as TGF-β, FGF-2, CTGF, and SDF-1 [16].

### 2.1. CAFs Promote PCa Cell Growth

**Figure 1:**
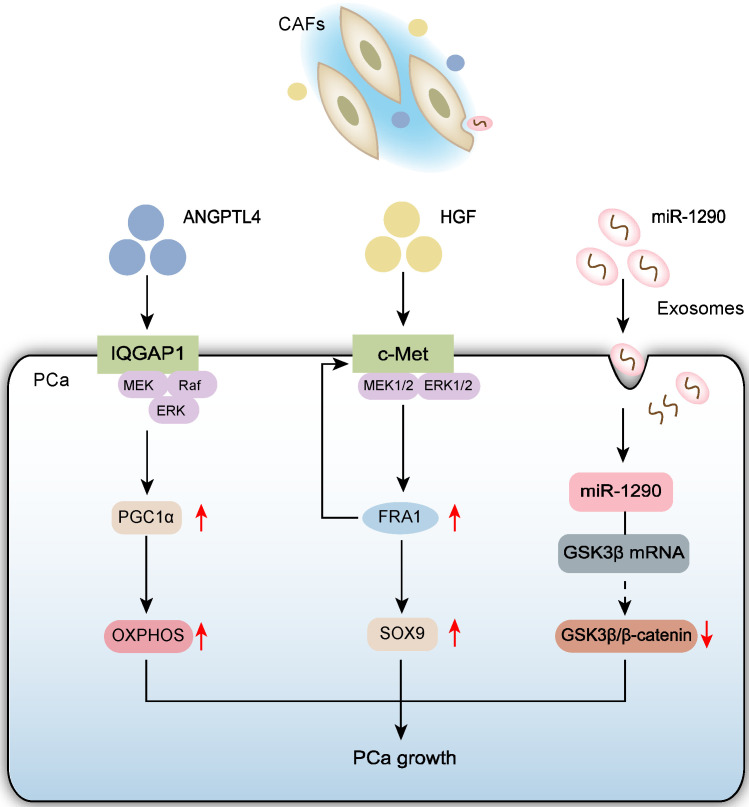
Schematic diagram depicts that CAFs drive the progression of PCa through three interconnected mechanisms: (1) ANGPTL4 secretion-mediated activation of the IQGAP1/ERK/PGC1α axis on PCa cell membranes enhancing mitochondrial biogenesis and oxidative phosphorylation function; (2) HGF secretion-triggered signaling that mediates the c-Met/ERK/FRA1 pathway upregulating SOX9 expression to promote tumor growth; (3) Exosomal miR-1290 delivery that inhibits the GSK3β/β-catenin signaling pathway in PCa cells.

Recent research highlights the pivotal regulatory role of CAFs in PCa progression. Through dual mechanisms of metabolic reprogramming and epigenetic regulation, CAFs establish a multi-dimensional regulatory network fostering tumor growth within the TME [17,18,19] (Figure 1). Specifically, CAFs precisely regulate PCa cell proliferation and growth both through paracrine release of various signaling molecules that activate downstream key pathways and via exosomal delivery of bioactive molecules. These findings provide new theoretical insights into the molecular mechanisms of stromal-epithelial cell interactions within the TME.

#### 2.1.1. CAFs Promote PCa Cell Proliferation via Paracrine Signaling

Metabolic reprogramming is significant in PCa initiation, progression, and metastasis. Consequently, CAFs and tumor metabolism are a focus. Alterations in mitochondrial dynamics, biogenesis, and metabolism are critical factors in numerous pathophysiological processes. The IQ motif-containing GTPase-activating protein 1 (IQGAP1) is expressed on PCa cell membranes. Angiopoietin-like protein 4 (ANGPTL4), primarily secreted by CAFs, is a multifunctional protein belonging to the angiopoietin family that plays key roles in various metabolic and non-metabolic diseases [20]. CAFs promote PCa growth via paracrine ANGPTL4: ANGPTL4 binds IQGAP1 on PCa cells, activating the ERK pathway and promoting PGC1α expression. This process enhances mitochondrial biogenesis and OXPHOS function [17].

Furthermore, compared to normal fibroblasts (NFs) and PCa cells, CAFs exhibit significantly enhanced secretion of hepatocyte growth factor (HGF). Studies show that HGF secreted by CAFs specifically upregulates SOX9 expression in PCa cells, a process essential for CAF-mediated tumor promotion. SOX9 is a key regulator of prostate epithelial lineage and an indispensable transcription factor during prostate organogenesis and carcinogenesis initiation [21]. Mechanistically, HGF regulates SOX9 expression by activating the c-Met receptor and its downstream MEK1/2-ERK1/2 signaling pathway. Further analysis revealed that the transcription factor FRA1, a key downstream effector of ERK1/2, directly mediates SOX9 transcriptional upregulation. Notably, FRA1 knockdown not only reduced SOX9 expression but also significantly inhibited phosphorylation of the c-Met receptor (at Tyr1234/1235), suggesting a potential positive feedback loop between the c-Met pathway and FRA1 expression. This finding is supported by clinical data: bioinformatic analysis shows a moderate positive correlation between MET gene expression and SOX9 levels. This study reveals a novel mechanism whereby CAFs regulate SOX9 expression via the HGF/c-Met-ERK1/2-FRA1 axis, playing an important role in PCa growth and progression. This discovery provides new theoretical insights into stromal-tumor cell interactions within the TME [18].

#### 2.1.2. CAF-Derived Exosomes Promote PCa Cell Growth

CAFs and cancer cells also interact via extracellular signaling mediated by exosomes. Exosomes are nano-sized vesicles secreted by cells that carry bioactive molecules like proteins, microRNAs (miRNAs), and lipids, playing crucial roles in intercellular communication [22]. Exosomes can transfer functional miRNA molecules from CAFs to cancer cells. Compared to exosomes from normal fibroblasts, exosomes from CAFs exhibit significantly elevated levels of miR-1290. GSK3β is a direct target of miR-1290 in PCa cells. MiR-1290 was initially identified in human embryonic stem cells and has been shown to play a crucial role in fetal neural development [23]. It is frequently overexpressed in a variety of human cancers, including colorectal cancer, breast cancer, gastric cancer, esophageal squamous cell carcinoma, pancreatic cancer, and lung cancer [24]. GSK3β is a serine/threonine kinase involved in diverse cellular processes including proliferation, the cell cycle, and metabolic pathways [25]. Exosomal miR-1290 secreted by CAFs promotes PCa cell growth and tumorigenesis by inhibiting the GSK3β/β-catenin signaling pathway [19].

### 2.2. CAFs Promote PCa Metastasis

**Figure 2:**
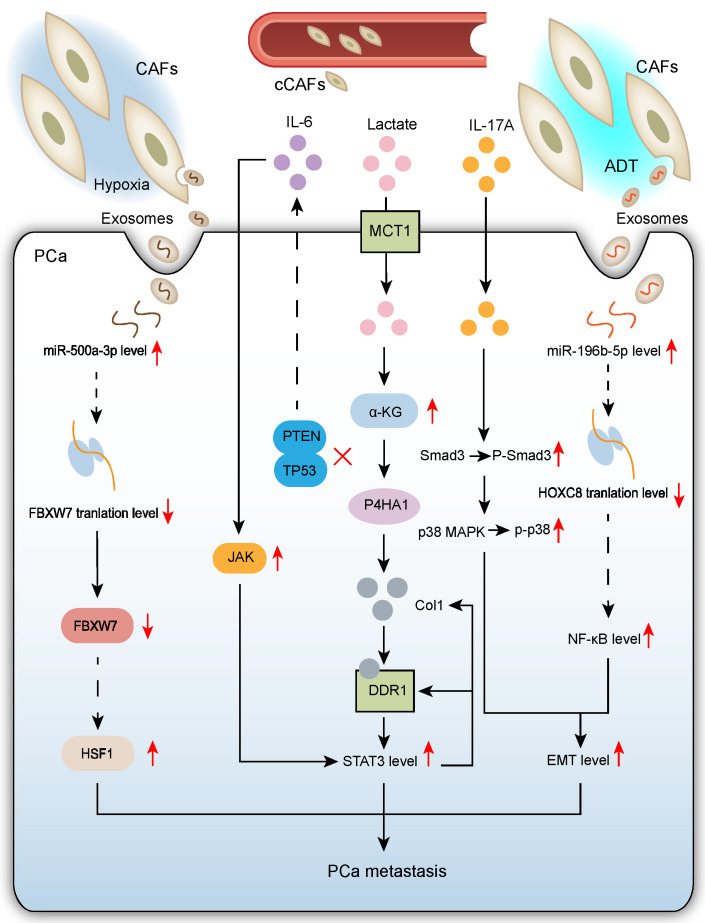
Schematic diagram depicts that CAFs drive PCa metastasis through four interconnected mechanisms: (1) Hypoxia-induced exosomal miR-500a-3p delivery that targets FBXW7/HSF1 signaling; (2) Lactate-mediated activation of the P4HA1/DDR1/STAT3 collagen-remodeling axis; (3) EMT promotion via IL-17A/Smad3/p38 MAPK secretion and castration-responsive exosomal miR-196b-5p/HOXC8/NF-κB signaling; (4) Synergistic crosstalk where p53-deficient tumor cells stimulate CAF-derived IL-6/Jak-Stat3 feedback loops, while cCAFs establish pre-metastatic niches as “soil” cells complementing CTC “seeds”.

Recent advances reveal the core regulatory role of CAFs in driving PCa metastasis through complex networks involving multiple pathways and targets [26,27,28,29,30,31] (Figure 2).

#### 2.2.1. CAF-Mediated Mechanisms Promoting PCa Metastasis in Hypoxic TME

Notably, rapid progression of PCa and other solid tumors is often associated with a hypoxic TME, a pathological feature significantly correlated with poor patient prognosis. However, existing research primarily focuses on the direct effects of hypoxia on tumor cells themselves, with less attention paid to its critical role in regulating CAF function within the PCa TME.

Clinical pathological analysis shows that primary lesions from metastatic PCa (mPCa) exhibit more pronounced hypoxia. Under hypoxic stimulation, exosomes secreted by CAFs significantly enhance the invasive and metastatic potential of PCa cells by delivering pro-metastatic factors.

Hypoxia induces significant upregulation of miR-500a-3p in CAF-derived exosomes. Upregulation of miR-500a has been observed in a range of malignancies, including chronic lymphocytic leukemia [32], breast cancer [33], hepatocellular carcinoma [34], and gastric cancer [35]. This microRNA, taken up by PCa cells, exerts pro-tumor effects by targeting and suppressing the tumor suppressor FBXW7. Notably, FBXW7, a well-established tumor suppressor gene, downregulates HSF1 expression upon overexpression, indicating that FBXW7 acts as a tumor suppressor in PCa cells by negatively regulating HSF1. Therefore, the hypoxia-induced CAF exosomal miR-500a-3p/FBXW7/HSF1 signaling axis constitutes an important molecular mechanism promoting PCa progression and metastasis [26].

ECM, a major component of the interstitium in multicellular tissues and organs, plays key roles in all biological processes by providing structural support, anchorage points for cell adhesion, water storage, diverse growth factors, and induction of intracellular signaling pathways [36]. Within the TME, the ECM acts as a critical structural component. Metabolites, particularly lactate, co-secreted by tumor and stromal cells within the ECM, promote the formation of invasive tumor phenotypes. Lactate is one of the most abundant metabolites in human circulation [37]. It is generated from the glycolytic end-product pyruvate via catalysis by lactate dehydrogenase. Under oxygen-sufficient conditions, pyruvate can be transported into mitochondria for biosynthetic pathways and ATP production; under hypoxia, pyruvate is converted to lactate [38]. However, pyruvate-to-lactate conversion can occur even with adequate oxygen. Within the PCa TME, CAFs are a major source of lactate secretion, significantly influencing malignant progression of cancer cells through metabolic reprogramming and epigenetic regulation. Lactate secreted by CAFs regulates collagen prolyl-4-hydroxylase (P4HA1) in PCa cells, maintaining collagen synthesis and modification. Specifically, lactate enters PCa cells via monocarboxylate transporter 1 (MCT1), increasing intracellular α-ketoglutarate (α-KG) levels. α-KG activates P4HA1. Experimental results show that silencing P4HA1 in PCa cells significantly reduced their lung retention efficiency compared to controls exposed to CAF-conditioned medium and lactate. This indicates P4HA1 is a key molecule mediating the initial critical step of metastatic colonization: organ-specific tumor cell retention. Meanwhile, discoidin domain receptor 1 (DDR1) is a non-integrin collagen receptor that specifically recognizes fibrillar collagen. DDR1 plays important roles in tumorigenesis, regulating tumor cell proliferation, migration, invasion, and energy metabolic reprogramming. Notably, DDR1 also modulates immune responses within the TME, playing a key role in tumor immune evasion [39]. DDR1 expression is significantly upregulated in metastatic PCa tissues and positively correlates with P4HA1 expression. Data reveal that DDR1 not only participates in collagen remodeling but, more importantly, mediates collagen sensing and downstream signal transduction during lactate-induced PCa metastasis. Specifically, DDR1 binding to type I collagen (Col1) triggers STAT3 pathway activation, which in turn regulates and sustains DDR1 and Col1 expression, forming a positive feedback loop. These findings collectively demonstrate that the P4HA1/DDR1/STAT3 signaling axis plays a key molecular regulatory role in promoting PCa metastasis [27].

#### 2.2.2. CAFs Enhance PCa Cell Epithelial–Mesenchymal Transition (EMT) to Promote Migration and Invasion

Within the PCa TME, CAFs significantly drive tumor cell migration, invasion, and metastasis through the secretion of key factors. Activation of the EMT program is a central mechanism. Delineating these signaling pathways provides new perspectives for understanding PCa progression and metastasis.

Studies show significantly higher IL-17A expression in PCa tissues compared to benign prostatic tissues. Mechanistic investigations reveal that CAFs directly secrete IL-17A, markedly enhancing the migration and invasion capabilities of PCa cells. This pro-tumor effect is primarily achieved by inducing EMT: EMT is a crucial phenotypic switch program where epithelial cells acquire mesenchymal characteristics, developing a mesenchymal phenotype with cancer stem cell-like features that ultimately promote tumor cell migration and invasion [40]. At the molecular level, IL-17A drives EMT by activating the Smad3/p38 MAPK signaling pathway [28]. Notably, IL-17A, a key member of the IL-17 cytokine family, is primarily secreted by Th17 cells under physiological conditions and participates in immune defense. However, aberrantly elevated IL-17A expression can become a “double-edged sword”, not only triggering autoimmune diseases but also promoting tumor development. This finding provides a new perspective for understanding PCa pathogenesis.

Furthermore, CAF-derived exosomes exhibit significantly enhanced pro-metastatic effects on PCa cells under castration conditions compared to androgen-sufficient environments [41]. During CRPC progression, CAFs secrete high levels of miR-196b-5p and deliver it to PCa cells via exosomes, promoting tumor cell migration and metastasis. MiR-196b-5p has been implicated in the pathogenesis of multiple malignancies, including lung cancer [42] and colorectal cancer [43]. Mechanistically, miR-196b-5p overexpression significantly enhances cancer cell EMT, migration, and invasion potential. Further molecular dissection revealed that miR-196b-5p functions by targeting and suppressing the homeobox gene HOXC8. Normally, HOXC8 can inhibit EMT by suppressing NF-κB pathway activation, while downregulation by miR-196b-5p relieves this inhibition, ultimately leading to EMT activation [29]. This discovery has important clinical implications: First, it elucidates the molecular mechanism by which CAFs promote metastasis after ADT via the exosomal miR-196b-5p/HOXC8/NF-κB axis, explaining the clinical phenomenon of metastasis post-ADT. Second, it suggests that targeting CAF-derived exosomes combined with ADT may be a novel strategy to improve outcomes in advanced PCa patients. However, two key scientific questions remain: The specific regulatory mechanism for miR-196b-5p upregulation in CAF exosomes post-ADT is unclear, and the precise molecular mechanism by which HOXC8 regulates the NF-κB pathway requires further elucidation. Addressing these questions will refine the molecular mechanism theory of PCa metastasis and provide new targets for developing more effective therapies.

Meanwhile, existing studies have demonstrated that pancreatic cancer cells [44] and colorectal cancer cells [45] can generate CAF-like cells through the EMT process. Although direct evidence of “cancer-derived CAFs” remains relatively limited in prostate cancer, epithelial cancer cells expressing EMT markers have been observed to migrate into the stroma and express fibroblast-specific genes in this malignancy [46]. This phenomenon is believed to contribute significantly to increased intratumoral heterogeneity, microenvironment complexity, and therapy resistance.

#### 2.2.3. Pro-Tumor Crosstalk Between Tumor Suppressor-Deficient Prostate Epithelial Cells and CAFs Drives PCa Metastasis

Earlier studies found frequent mutations in tumor suppressor genes PTEN and TP53 in PCa, serving as predictors of early metastatic dissemination and poor prognosis [47]. In-depth mechanistic studies show that P53 deletion triggers pro-tumorigenic interactions between prostate epithelial cells (PECs) and CAFs. Notably, while P53 loss does not affect early proliferation or prostate intraepithelial neoplasia (PIN) formation in PTEN-deficient epithelial cells, nor later growth arrest and senescence, it significantly induces epithelial cell plasticity, promoting adenocarcinoma formation and accelerating metastatic spread.

The core mechanism involves P53 deletion establishing a unique TME regulatory network: On one hand, P53-deficient epithelial cells non-cell-autonomously stimulate CAFs to produce IL-6; on the other hand, IL-6 secreted by CAFs further enhances JAK/STAT3 signaling activity in epithelial cells through a positive feedback loop. This feedforward loop mechanism drives the acquisition of epithelial cell plasticity, contrasting with previously reported autocrine/paracrine epithelial signaling mechanisms. Critically, TRP53-deficient tumors send specific signals stimulating CAFs to enhance IL-6 secretion, revealing a novel mechanism where P53 loss promotes PCa progression by inducing pro-tumorigenic crosstalk between PTEN-deficient PECs and CAFs [30].

However, a key question remains regarding the specific cellular interaction mechanism in this network: Do PECs directly act on CAFs to enhance IL-6 secretion, or do they achieve this indirectly by regulating other TME cells (e.g., immune or endothelial cells)? Clarifying this will provide a more comprehensive understanding of the TME regulatory mechanisms underlying PCa metastasis.

#### 2.2.4. Circulating CAFs (cCAFs) Emerge as a Novel Mechanism Promoting Cancer Progression and Metastasis

Interestingly, the circulatory system of PCa patients contains not only circulating tumor cells (CTCs) but also active CAFs. A study first identified functional fibroblast activation protein (FAP)-positive cCAFs in the peripheral blood of metastatic castration-resistant prostate cancer patients, classifying them into CD45+ and CD45− subpopulations based on CD45 expression. Using single-cell RNA sequencing, three distinct CAF subtypes were systematically identified in PCa: 1. Myofibroblast-like CAFs (myCAFs): High expression of activated fibroblast markers and ECM-related genes. 2. Inflammatory-like CAFs (iCAFs): Significant upregulation of inflammatory factors. 3. Antigen-presenting CAFs (apCAFs): Specific high expression of antigen presentation-related molecules. Further analysis confirmed the mesenchymal origin of isolated cCAFs by positive vimentin staining. Functional assays confirmed their ability to synthesize ECM proteins (e.g., Collagen I) and respond to TGFβ stimulation. Based on these characteristics, the authors inferred that the cCAFs identified in mPCa patients primarily belong to the myCAF subtype.

The study proposed a novel perspective on “seed-soil” interactions: During metastasis, cCAFs act as “soil” cells responsible for establishing the pre-metastatic niche, while CTCs act as “seed” cells that ultimately form metastatic lesions. These two circulating cell populations promote cancer progression and metastasis through distinct yet complementary mechanisms [31]. Moreover, in breast cancer research, although treatment with liposomal doxorubicin (L-Dox) significantly suppressed tumor growth, it led to a marked increase in the number of CTCs and cCAFs in the blood, suggesting that chemotherapy may facilitate tumor dissemination [48]. Furthermore, cCAFs have been detected in the peripheral blood of patients not only with breast cancer but also with colorectal and prostate cancers [49], further supporting the notion that cCAFs may represent a widespread phenomenon associated with metastasis across multiple cancer types.

### 2.3. CAFs Promote Chemoresistance to Taxanes in PCa Cells

For metastatic PCa patients, taxane-based chemotherapy is a first-line treatment option [50]. CAF-derived exosomes and cytokines play key roles in promoting chemoresistance in PCa. CAF-derived exosomes reduce the chemosensitivity of parental PCa cells and enhance drug resistance in resistant cells. CAF-secreted exosomes are enriched in miR-423-5p. MiR-423-5p plays diverse roles in tumorigenesis and cancer progression [51,52], and has also emerged as a potential key player in brain metastasis [53]. This miRNA inhibits the expression of GREM2 (a bone morphogenetic protein antagonist) by regulating the TGF-β signaling pathway, thereby reducing PCa cell sensitivity to taxanes. Specifically, exosomally delivered miR-423-5p promotes tumor cell survival via the TGF-β/GREM2 axis [54]. Recent research highlights the critical role of ferroptosis in disease progression and therapy resistance in various cancers [55]. Ferroptosis is an iron-dependent form of cell death characterized by excessive lipid peroxidation and accumulation of reactive oxygen species (ROS) [56]. In PCa treatment, ferroptosis regulation is closely linked to chemoresistance. CAF-derived exosomes can suppress ferroptosis in PCa cells through specific mechanisms. Specifically, CAF exosomes inhibit erastin-induced lipid ROS accumulation and mitigate mitochondrial damage caused by erastin. Further mechanistic investigation identified exosome-carried miR-432-5p as the key effector. This miRNA targets and suppresses ChaC glutathione specific gamma-glutamylcyclotransferase 1 (CHAC1) expression, reducing intracellular glutathione (GSH) depletion, thereby maintaining glutathione peroxidase 4 (GPX4) activity and ultimately inhibiting lipid peroxide accumulation and ferroptosis. Notably, the chemotherapy drug docetaxel (DTX) stimulates CAFs to increase miR-432-5p secretion, forming a positive feedback loop. Therefore, CAF-derived miR-432-5p not only inhibits ferroptosis but also promotes PCa cell proliferation and reduces sensitivity to DTX [57].

Additionally, ANGPTL4 secreted by CAFs promotes mitochondrial biogenesis in PCa cells by upregulating PGC1α expression, enhancing OXPHOS function. ANGPTL4 interacts with IQGAP1 located on PCa cell membranes, triggering activation of the cRAF-MEK-ERK-PGC1α signaling cascade. PCa cells with enhanced OXPHOS exhibit reduced sensitivity to DTX, confirming that enhanced mitochondrial OXPHOS inhibits chemosensitivity in PCa [17].

**Figure 3:**
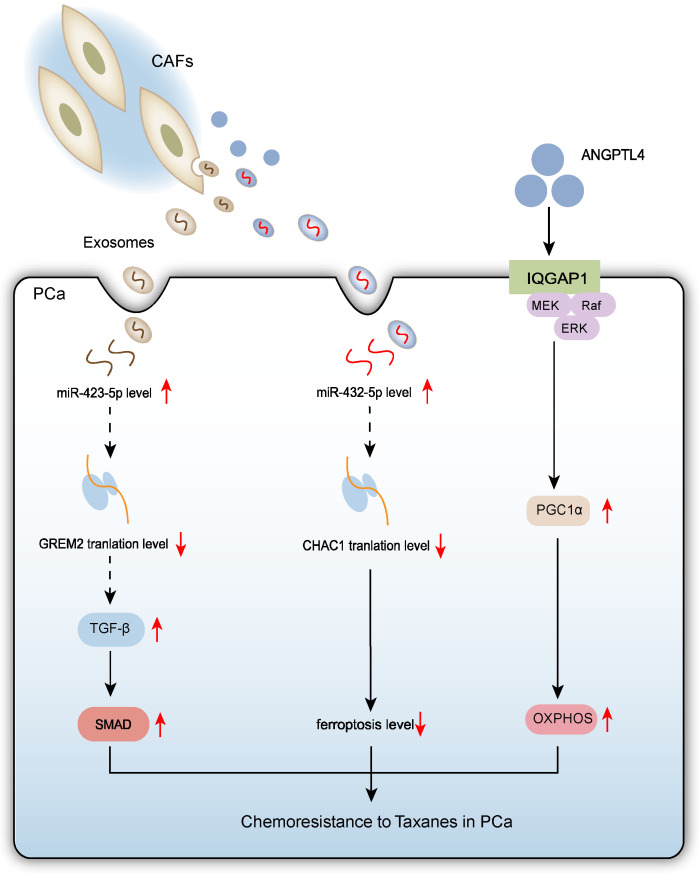
Schematic diagram depicts that CAFs confer taxane resistance in PCa cells through three interconnected mechanisms: (1) Exosomal miR-423-5p delivery that suppresses the TGF-β/GREM2 signaling axis; (2) Exosomal miR-432-5p-mediated inhibition of ferroptosis by targeting CHAC1; (3) ANGPTL4 secretion-mediated activation of the IQGAP1/ERK/PGC1α axis on PCa cell membranes enhancing mitochondrial OXPHOS function.

All three mechanisms involve CAFs reducing PCa cell chemosensitivity through paracrine signals (exosomes or secreted proteins), involving metabolic or signaling pathway alterations (TGF-β/GREM2 axis inhibition, ferroptosis suppression, OXPHOS enhancement), allowing tumor cells to adapt to chemotherapy pressure. These changes ultimately lead to taxane (e.g., DTX) resistance. (Figure 3)

## 3. CAFs Promote the Development and Progression of CRPC

Hormone therapy initially controls PCa by inhibiting the androgen receptor (AR) signaling pathway but ultimately leads to the development of CRPC [58]. Multiple mechanisms promote tumor recurrence by restoring AR signaling or bypassing AR dependence, allowing PCa growth. Increasing evidence indicates that the TME plays a key role in CRPC.

### 3.1. Different CAF Subtypes Promote CRPC

As mentioned earlier, in prostate cancer, CAFs can be categorized into three distinct subtypes: myCAFs, iCAFs and apCAFs. This heterogeneity gives rise to fundamental questions regarding the potential interconversion between CAF subtypes or, alternatively, the stability of their respective phenotypes. Current evidence suggests that fibroblast states are highly plastic. During tissue remodeling, different fibroblast subpopulations are not only regulated in an ECM-dependent manner but also exhibit functional diversity [59]. Given that cancer is often conceptualized as a “wound that does not heal” [60], it is plausible that analogous regulatory mechanisms and comparable functional specialization of fibroblasts may occur within the tumor microenvironment.

Meanwhile, a novel CAF subtype, specific to CRPC, has been identified and is characterized by high expression of HSD17B2. This subtype exhibits inflammatory features. Within the CRPC TME, iCAFs act as key effector cells in intercellular interactions, demonstrating stronger cellular communication capabilities via specific ligand-receptor pairs. Compared to hormone-sensitive PCa (HSPC), HSD17B2 expression is significantly elevated in CRPC-associated fibroblasts, while it is barely detectable in CAFs from HSPC. Notably, high HSD17B2 expression in CAFs may be closely linked to the development of resistance to hormone therapy. Mechanistically, HSD17B2 catalyzes the inactivation of dihydrotestosterone (DHT) in CAFs, thereby suppressing AR transcriptional activity and ultimately leading to upregulation of ITGBL1 expression [61]. Previous studies have confirmed that ITGBL1 promotes tumor cell invasion and migration by activating the NF-κB signaling pathway in PCa cells [62]. The AR-ITGBL1 signaling axis in CAFs promotes PCa cell migration, invasion, and the acquisition of a castration-resistant phenotype, providing new theoretical insights into CRPC progression.

Previous research largely focused on the autocrine effects of secreted phosphoprotein 1 (SPP1) on PCa cells, overlooking the SPP1-ERK paracrine mechanism driving castration resistance. ADT-induced SPP1-expressing myofibroblastic CAFs (SPP1myCAFs) are key stromal components driving CRPC development. SPP1myCAFs originate from iCAFs in HSPC. Androgen receptor inhibition drives the conversion of iCAFs to SPP1myCAFs via activation of the TGF-β signaling pathway. TGFβ-induced SOX4 expression orchestrates this CAF conversion through SWI/SNF complex-dependent chromatin remodeling. SPP1myCAFs subsequently confer ADT resistance to PCa via an SPP1-ERK paracrine mechanism [63].

The above studies confirm the roles of myCAFs and iCAFs in CRPC progression. The role of apCAFs in this context remains largely unknown and warrants further investigation.

### 3.2. CAFs Promote CRPC via Paracrine Mechanisms

During PCa progression, ADT or AR loss inactivates the AR signaling pathway, thereby relieving its inhibitory effect on LIM domain only 2 (LMO2) expression in stromal fibroblasts. This leads to the conversion of normal prostate fibroblasts to CAFs or LMO2 overexpression upon ADT-induced AR inactivation. Studies show that upregulated stromal LMO2 secretes cytokines like FGF-9 and IL-11 via a paracrine mechanism. These cytokines activate downstream oncogenic signaling pathways, ultimately reactivating AR signaling through non-androgen-dependent phosphorylation. This molecular mechanism elucidates the central role of LMO2 and its induced cytokines in driving non-cell-autonomous PCa progression and castration resistance [64].

Although second-generation anti-androgens like enzalutamide (Enz) significantly prolong survival in CRPC patients and exhibit potent AR signaling inhibition [65], even the most effective AR-targeted therapies fail to achieve complete remission, with patients inevitably developing Enz resistance [66]. Studies found significantly upregulated expression of the immunomodulatory chemokine CCL5 in CAFs compared to NFs. CAF-derived CCL5 promotes AR upregulation in PCa cells via paracrine action, mediating Enzalutamide resistance. Furthermore, CCL5 upregulates PD-L1 expression on tumor cells, inducing immune evasion [67]. Mechanistically, CCL5 binds to the CCR5 receptor on tumor cell surfaces, activating the downstream AKT signaling pathway, which cooperatively promotes AR and PD-L1 upregulation, ultimately driving CRPC progression and therapy resistance.

Both mechanisms involve paracrine regulation between stromal cells and PCa cells within the TME, emphasizing the role of non-cell autonomy in PCa progression. Both LMO2 and CCL5 promote tumor cell AR signaling reactivation via paracrine signaling. However, LMO2 focuses more on growth factor-mediated non-canonical AR activation, while CCL5 directly regulates AR via AKT and additionally impacts the immune microenvironment.

## 4. Anti-Tumor Effects of CAFs in PCa

While most studies report pro-tumorigenic functions of CAFs, accumulating evidence suggests that certain CAF subtypes may also exert anti-tumor effects [68].

The desmoplastic reaction is fundamentally a host defense mechanism, biologically analogous to physiological wound healing and tissue regeneration. This reaction might delay or even block the malignant transformation to invasive carcinoma by repairing damaged tissue or creating physical barriers, a phenomenon observed in pancreatic ductal adenocarcinoma (PDAC).

Notably, the desmoplastic stroma during cancer progression exhibits dynamic evolution: its heterogeneous cellular components (e.g., myofibroblasts) and non-cellular components (e.g., ECM) undergo adaptive remodeling alongside the evolving genomic features of cancer cells. Although substantial evidence indicates that the accumulation of αSMA-positive myofibroblasts and type I collagen deposition (core features of tumor fibrosis) typically promote tumor growth in PDAC and other solid tumors [69], paradoxically, experimental depletion of these myofibroblasts worsened patient prognosis. This paradox suggests that myofibroblast- and collagen I-mediated fibrotic reactions may primarily exert protective effects, rather than the traditionally perceived pro-tumor effects, at different stages (particularly early vs. late) of PDAC development [44]. This raises a key scientific question: Do specific CAF subtypes exert anti-tumor effects at specific stages during PCa progression?

Based on functional characteristics, CAFs can be classified into pro-tumorigenic ECM-associated CAFs (ECM-CAFs) and anti-tumor lymphocyte-associated CAFs (Lym-CAFs). Lym-CAFs likely emerge when stromal fibroblasts “hijack” or “mimic” the gene expression program of immune cells, thereby acquiring a distinctive “immune-cell-like” phenotype [70]. There is also evidence that myeloid cells may undergo transdifferentiation or EMT-like processes to adopt fibroblastic features [71]. Nonetheless, it is widely accepted that the activation of local tissue-resident fibroblasts remains the predominant source of CAF generation. ECM-CAFs highly express markers like CD248, COL1A1, COL1A2, CNN1, and CCN2, while Lym-CAFs are significantly enriched in cytokines like TNFAIP6, IL33, and CXCL8-11. The formation of these CAF subsets is regulated by different cytokines: TGFβ stimulation induces upregulation of ECM-CAF markers, forming “induced ECM-CAFs” (i-ECM-CAFs); TNFα/IFNγ stimulation promotes Lym-CAF marker expression, generating “induced Lym-CAFs” (i-Lym-CAFs). At the molecular level, YAP1 and NF-κB p65 are the core transcription factors regulating these two CAF phenotypes. YAP1 is highly expressed in ECM-CAFs, and its overexpression enhances the ECM-CAF phenotype. Conversely, silencing YAP1 in i-LymCAFs promotes the Lym-CAF phenotype. Mechanistically, YAP1 suppresses NF-κB p65 activation by directly interacting with IKKα and inhibiting its phosphorylation. Notably, NF-κB p65, a key transcription factor for Lym-CAFs, is negatively regulated by YAP1; YAP1 maintains the ECM-CAF phenotype by inhibiting p65 nuclear translocation. Functionally, ECM-CAFs support tumor progression by promoting collagen deposition, while Lym-CAFs exert anti-tumor effects by recruiting and activating CD8+ T cells [72]. This functional divergence stems from their different induction mechanisms: TGFβ-induced ECM-CAFs exhibit pro-tumorigenic properties via excessive ECM secretion, while TNFα/IFNγ-induced Lym-CAFs establish an anti-tumor microenvironment via cytokine secretion. Based on these findings, targeting YAP1 holds therapeutic potential for CAF phenotype conversion: Selective ablation of YAP1 in ECM-CAFs could drive their transformation towards anti-tumorigenic Lym-CAFs, thereby enhancing the efficacy of anti-PD-1 antibody therapy in PCa.

This research prompts a fundamental shift in our understanding of the TME: CAFs are not a monolithic pro-tumor entity but comprise subpopulations with opposing functions. For PCa, investigating the existence of protective subpopulations like Lym-CAFs and their stage-specific roles is crucial. More importantly, the strategy of targeting YAP1 to drive CAF phenotype switching from pro-tumor to anti-tumor offers a highly attractive new avenue for overcoming the immunosuppressive TME in PCa and improving immunotherapy response.

## 5. Predicting PCa Patient Prognosis Using CAF-Associated Genes and Expression Products

Currently, the AR is one of the most intensively studied and therapeutically targetable oncogenes in PCa. It is frequently amplified and/or mutated in metastatic disease, promoting tumorigenesis by regulating the androgen-dependent transcription of growth-related genes [73]. Concurrently, CAFs promote tumor initiation, growth, invasion, and metastasis through multiple signaling pathways and paracrine mechanisms, including exosomal delivery. This suggests that analyzing CAF-associated genes and their expression products could provide important insights for predicting PCa patient prognosis and guiding treatment decisions. However, CAFs exhibit significant heterogeneity in cellular origin, biomarker expression, and functional properties [74], posing substantial challenges for comprehensively assessing their prognostic value.

To overcome this challenge and deepen the understanding of CAF roles, researchers integrated transcriptomic data from CAFs and PCa TCGA cohorts to identify a set of CAF-derived gene signatures with predictive value for poor prognosis. Functional pathway enrichment analysis revealed that these CAF-associated genes (including IL13RA2, GDF7, IL33, CXCL1, TNFRSF19, CXCL6, LIFR, CXCL5, IL7, TSLP, and TNFSF15) are significantly enriched in the “cytokine-cytokine receptor interaction” pathway. Intriguingly, a specific subgroup of PCa patients with cumulative low expression of these genes had significantly lower survival rates than patients with high expression [75]. This seemingly paradoxical phenomenon suggests that within the complex ecosystem of the TME, focusing solely on the prognostic value of individual genes within a single cell type may be limited. A more accurate understanding requires expanding the research perspective to the transcriptional landscape of different cell populations within tumor tissue and fully considering the intricate multidirectional interaction networks between tumor cells and TME components.

Furthermore, researchers identified a CAF subtype termed CTSK+ MRC2+ CAF-C1, associated with potential antigen-presenting functions. ADT significantly reduced the number of antigen-presenting CAFs. Specifically, after castration, the CTSK+ MRC2+ CAF-C1 subtype downregulated pathways related to antigen presentation and processing. To understand the impact of androgen deprivation, CAF cell lines were treated with ADT in vitro, and the expression changes in characteristic genes were examined. Results showed that after ADT treatment, THBS2, COL5A1, and MARCKS were further significantly upregulated, while DPT showed a non-significant downregulation trend. A 4-gene prognostic model (APPCAFRS) was constructed based on apCAFs, with core genes THBS2, DPT, COL5A1 and MARCKS (where DPT is protective and the others are risk genes) [76]. The risk score of this model negatively correlated with the infiltration levels of various immune cells. A high-risk score significantly predicted poor prognosis and unfavorable clinical outcomes in PCa patients, independent of clinical indicators like Gleason grade, PSA level, and T stage.

The biological mechanism underlying the paradoxical finding that low expression of certain CAF subtype-associated genes correlates with worse prognosis remains incompletely understood. Is this due to altered functional states of specific CAF subpopulations, compensatory responses from other TME cells (e.g., immune cells), or other factors? Furthermore, CAF subtype classification is not exhaustive, and the interactions between different subtypes need clarification. The mechanism and functional significance of DPT downregulation, as a protective gene, warrant particular attention. While the APPCAFRS demonstrated good prognostic value in the study, whether the expression of its component genes can be stably detected in clinical samples (e.g., liquid biopsies) is also a key issue for future translation.

## 6. Novel CAF-Targeting Therapeutic Strategies for PCa

The pro-tumorigenic mechanisms of CAFs within the TME have emerged as new targets to overcome therapy resistance in PCa. However, developing therapies targeting CAFs is complex due to their existence as heterogeneous populations containing both pro-tumorigenic and anti-tumorigenic subpopulations with significantly contradictory functions [77]. Moreover, the key signaling networks governing CAF phenotype switching are not fully resolved, and some emerging CAF-targeting strategies still face significant gaps in clinical translation.

First, Erdafitinib, a selective pan-FGFR inhibitor, has demonstrated clinical value [78]. Approved for treating advanced bladder cancer with FGFR mutations, it is also undergoing clinical trials for advanced solid tumors like PCa and metastatic breast cancer. Therefore, exploring strategies to enhance Erdafitinib efficacy is clinically significant. S100 calcium-binding protein A11 (S100A11), a typical EF-hand calcium-binding protein of the S100 family, has been implicated in tumor initiation, progression, and metastasis by multiple studies [79]. Research found that S100A11 knockout significantly enhanced the inhibitory effect of Erdafitinib on CAFs. Notably, combined knockout of S100A11 in both tumor cells and CAFs produced a more pronounced therapeutic effect than knockout in tumor cells alone. Specifically, in RM-1 cell and CAF models, S100A11 knockout combined with Erdafitinib treatment effectively suppressed CAF activity, significantly increased infiltration of CD4+ T cells and effector CD8+ T cells within the TME and markedly reduced tumorigenic capacity [80]. The therapeutic effect of combined S100A11 knockout in both tumor cells and CAFs was significantly superior to knockout in tumor cells alone. These findings provide a new potential target for PCa therapy: targeting S100A11 to enhance the efficacy of existing FGFR inhibitors.

Notably, besides targeting tumor cell-CAF interactions, directly modulating CAF phenotypes is also an important strategy. Studies found that patient-derived CAFs, compared to matched normal prostate fibroblasts (NPFs), exhibit significant upregulation in their gene expression profiles of gene sets related to cardiomyopathy and cation channel activity, including genes encoding sodium, calcium, and potassium channels [81]. These ion channels are not only key regulators of specific physiological processes like muscle contraction but also play important roles in biological processes commonly dysregulated in cancer cells and CAFs, such as the cell cycle, survival, migration, and invasion [82]. Based on the similarity in ion channel expression between CAFs and contractile cells (e.g., cardiomyocytes), researchers proposed an innovative strategy: utilizing antiarrhythmic drugs (acting as cation channel blockers) to revert activated CAFs to a tumor-suppressive normal state (“normalization”), thereby reshaping the TME and inhibiting tumor growth [83]. Experiments confirmed that specific calcium channels (CACNA1H, CACNB1, CACNB3), sodium channels (SCN2A, SCN1B), and potassium channels (KCNS3) are expressed at higher mRNA and/or protein levels in CAFs than in NPFs, indicating cation channel upregulation is a feature of CAF activation. Functional studies showed that antiarrhythmic drugs (e.g., the calcium channel blocker nifedipine, the sodium channel blocker flecainide) effectively impaired the pro-tumorigenic capabilities of CAFs, specifically by inhibiting migration, affecting matrix remodeling, blocking pro-tumorigenic factor release (e.g., IL-8), reversing transcriptional profiles (towards a state closer to NPFs), and weakening pro-tumorigenic effects in vivo. Future animal studies are needed to evaluate the effective dose window of antiarrhythmic drugs for impairing CAF pro-tumorigenic capabilities in vivo and to explore potential dose-dependent toxicity and adverse effects. This will provide crucial pharmacological and safety foundations for subsequent exploration of the clinical translation path for these drugs in PCa treatment.

Expanding the CAF-targeting approach further, eliminating specific pro-tumorigenic CAF subpopulations shows unique value. Fibroblast activation protein alpha (FAP), a transmembrane prolyl protease, is highly expressed on the surface of highly pro-tumorigenic CAFs in the stroma of almost all epithelial-derived tumors [84]. Eliminating these pro-tumorigenic CAFs disrupts interactions between components within the TME, inducing cancer cell death and promoting immune cell infiltration [85]. To achieve specific elimination of FAP-positive CAFs, researchers constructed an antibody-drug conjugate, linking the anti-FAP antibody huB12 to the cytotoxic payload monomethyl auristatin E (MMAE) (huB12-MMAE). After targeted elimination of CAFs using huB12-MMAE, the levels of secreted IL-6, IL-8, and CSF1 in their supernatants significantly increased. While these factors are known to mediate pro-angiogenic and tumor-promoting processes in the TME (e.g., IL-6 and IL-8 can upregulate VEGFA expression in tumor cells to drive angiogenesis) [86], analysis of 22Rv1 tumor cells under huB12-MMAE treatment showed no change in VEGFA expression. Instead, a significant increase in CXCL11 and PD-L1 mRNA levels was observed in 22Rv1 cells. CXCL11 has been shown to recruit CD8+ T cells for tumor cell killing [87], while the traditional view suggests that downregulating PD-L1 in PCa exerts anti-tumor effects [88]. Synthesizing these findings, the increase in IL-6, IL-8, and CSF1 levels in the TME upon CAF clearance in the huB12-MMAE context primarily drives the formation of a pro-inflammatory microenvironment rather than directly promoting angiogenesis or tumor proliferation [89]. Therefore, the mechanism of FAP-directed therapies like huB12-MMAE likely involves modulating the immune microenvironment, inducing a pro-inflammatory response, and promoting its targeted attack on the tumor. However, a limitation is that the precise molecular signaling pathways mediating this immune microenvironment modulation and pro-inflammatory response induction by huB12-MMAE therapy remain to be elucidated.

Utilizing immunomodulators to directly reprogram CAF function and relieve their suppression of T cells also shows promise for enhancing anti-tumor immune responses. Extracted from Lentinus edodes, the polysaccharides MPSSS reprograms CAFs by activating the TLR4-NF-κB pathway, effectively relieving CAF-mediated suppression of T cells and thereby enhancing anti-tumor immune responses [90]. Its mechanism involves significantly reducing the expression of the CAF activation marker α-SMA in a dose-dependent manner, suggesting MPSSS may drive CAFs towards a quiescent state. Based on this immunomodulatory effect, combining MPSSS with direct tumor-killing drugs holds promise for overcoming tumor immune escape and improving comprehensive therapeutic efficacy. Interestingly, Ligustilide exhibits similar effects [91]. However, the direct interaction mechanisms between CAFs and T cells require further elucidation, and in vivo anti-tumor efficacy needs validation through subsequent studies.

Nanomedicines offer a promising new avenue for cancer therapy due to their potential to enhance drug specificity, improve pharmacokinetic properties, and reduce adverse effects [92]. Nanoparticles (NPs) sized 100–200 nm can preferentially accumulate in tumors via the enhanced permeability and retention effect [93], but their deep penetration within tumor tissue is limited. A key barrier is the stromal barrier formed by CAFs, severely hindering NP penetration and limiting nanodrug efficacy. In contrast, small-sized NPs (<50 nm) offer superior tumor distribution but require addressing circulation stability and targeting issues. To tackle this, researchers developed a CAF-triggered transformable nanosystem (HA@DSP-pep-DSP) [94]. Its core design includes a peptide sequence (Asp-Ala-Thr-Gly-Pro-Ala) specifically cleavable by the FAP-α enzyme overexpressed on CAF surfaces. The initial particle size is approximately 200 nm with a negative charge, favoring stable blood circulation. Upon reaching the tumor site and contacting CAFs, FAP-α cleaves the peptide chain, causing NP dissociation into ~20 nm small particles. These small particles expose a positive charge surface, effectively penetrating the dense tumor stroma and promoting uniform drug distribution within the tumor core. For drug delivery, the anticancer drug doxorubicin (DOX) is conjugated to the nanocarrier via disulfide bonds. In the high glutathione (GSH) environment inside tumor cells or CAFs, the disulfide bonds are reductively cleaved, enabling specific DOX release. The released DOX acts on both tumor cells and CAFs, significantly reducing expression of CAF markers (α-SMA, FAP-α, TGF-β), thereby weakening the physical stromal barrier and inhibiting pro-cancer signaling.

CAF-targeting therapeutic strategies need to integrate simple elimination with precise modulation. By reversing their pro-tumorigenic phenotype, dismantling the physical stromal barrier, and reshaping the immunosuppressive microenvironment, breakthrough therapies for advanced PCa may emerge (Table 1). Future efforts require integrating multi-omics analysis, intelligent nanotechnology, and immune engineering approaches to ultimately achieve clinical translation of personalized combination regimens.

## 7. Conclusions and Future Directions

This review systematically elucidates the central driving role of CAFs in PCa cell proliferation, invasion, metastasis, chemoresistance, and CRPC development through complex mechanisms including paracrine signaling, exosomal delivery, and metabolic products. It also highlights the high heterogeneity of CAFs, distinguishing pro-tumorigenic subtypes from potentially anti-tumorigenic ones, and emphasizes that their functional state is dynamically regulated by TME signals and epithelial cell genomic status. Molecular signatures based on CAFs show promise as prognostic biomarkers for PCa. More importantly, targeting CAFs and the signaling networks they mediate represents a promising frontier for overcoming current therapeutic bottlenecks and developing novel PCa therapies. However, significant challenges remain: The specific functions of different CAF subtypes at distinct stages of PCa (especially early vs. late) and their dynamic transition mechanisms require deeper investigation. The role and regulatory mechanisms of anti-tumor CAF subpopulations in PCa are poorly understood. The optimal timing window and therapeutic strategy for targeting CAFs need precise selection based on disease stage and CAF heterogeneity. Efficient delivery of targeted therapeutics through dense tumor stroma remains a major hurdle.

In summary, CAFs emerge as master regulators not only of PCa progression but also of therapeutic resistance. Their substantial heterogeneity and inherent plasticity pose significant research challenges yet simultaneously reveal a rich landscape of novel therapeutic targets and intervention strategies. The paradigm shift from broad stromal ablation towards the precision modulation of specific CAF subpopulations and their functions, when rationally integrated with established and emerging anti-tumor modalities, represents a promising therapeutic avenue to overcome treatment resistance and improve outcomes for patients with advanced and CRPC. Future research imperatives include elucidating CAF biology at unprecedented resolution, developing specific and potent modulators, and strategically incorporating these advancements into the clinical management framework for prostate adenocarcinoma.

## Figures and Tables

**Table 1:** Targets for CAFs, inhibitors and effects.

Target	Inhibitor(s)	Effects on CAFs/TME	Reference
ANGPTL4-IQGAP1	QGGP	Inhibits CAF-induced chemoresistance, enhances docetaxel sensitivity, promotes IQGAP1 degradation	[17]
HGF/c-Met	Capmatinib	Blocks CAF-induced SOX9 upregulation, inhibits tumor migration, invasion and stemness, reverses CAF pro-tumor effects	[18]
MEK1/2, ERK1/2	U0126 (MEK1/2 Inhibitor), SCH772984 (ERK1/2 Inhibitor)	Inhibits HGF/CAF-induced SOX9 expression, reduces tumor invasion	[18]
miR-1290	miR-1290 antagomir	Inhibits PCa cell migration, invasion, stemness and EMT, reverses CAF exosome pro-metastatic effect, activates GSK3β/β-catenin signaling	[19]
Exosome secretion	GW4869	Blocks internalization of CAF exosomes by PCa cells, inhibits CAF exosome-mediated promotion of migration and invasion	[26]
MCT1	AR-C155858	Blocks lactate uptake from CAF secretion, inhibits P4HA1 activity and collagen deposition	[27]
P4HA1	DHB	Inhibits collagen hydroxylation, reduces tumor cell invasion and lung colonization capacity	[27]
DDR1	7rh	Blocks collagen-DDR1 signaling axis, inhibits STAT3 phosphorylation, stemness and transendothelial migration	[27]
STAT3	Stattic	Inhibits STAT3 phosphorylation, disrupts lactate-P4HA1-COL1-DDR1 positive feedback loop, reduces invasion and prostasphere formation	[27]
p38 MAPK	SB203580	Inhibits p38 phosphorylation, reverses EMT and cell invasion	[28]
NF-κB	BAY 11-7082	Blocks P65 nuclear translocation, reverses miR-196b-5p-induced EMT	[29]
JAK	Ruxolitinib	Inhibits STAT3 phosphorylation, blocks CAF-induced EMT	[30]
TGF-β	LY2109761	Partially reverses CAF exosome-induced chemoresistance, inhibits PCa cell proliferation/colony formation, promotes apoptosis	[54]
ERK	GDC-0994	Inhibits ERK downstream phosphorylation of p90RSK, blocks SPP1-mediated paracrine resistance, delays CRPC progression	[63]
IL-11RA	BMPP-11	Targets IL11RA, inhibits PCa tumor growth	[64]
CCR5	Maraviroc	Blocks CCL5-CCR5 axis, reduces AR/PD-L1 expression, enhances enzalutamide efficacy, inhibits tumor growth, reverses immune evasion	[67]
FGFR	Erdafitinib	Inhibits PI3K/AKT pathway, reduces CAF activity, combined with S100A11 knockdown enhances T-cell infiltration, reduces tumor volume	[80]
IL-8, GIF	Flecainide/Nifedipine	Reduces secretion of pro-metastatic factors IL-8 and GIF, reverses CAF-induced PCa cell migration and EMT	[83]
α-SMA	Ligustilide/MPSSS	Downregulates α-SMA expression, reduces CAF activity, reverses immunosuppressive phenotype	[90,91]

## Data Availability

Data sharing is not applicable to this article as no datasets were generated or analyzed during the current study.
